# Improved stacking ensemble learning based on feature selection to accurately predict warfarin dose

**DOI:** 10.3389/fcvm.2023.1320938

**Published:** 2024-01-19

**Authors:** Mingyuan Wang, Yiyi Qian, Yaodong Yang, Haobin Chen, Wei-Feng Rao

**Affiliations:** ^1^ Department of Pharmacy, Fuwai Yunnan Cardiovascular Hospital, Kunming, China; ^2^ School of Mechanical Engineering (Shandong Institute of Mechanical Design and Research), Qilu University of Technology (Shandong Academy of Sciences), Jinan, Shandong, China; ^3^ Department of Pathology, Qujing First People's Hospital, Qujing, Yunnan, China

**Keywords:** warfarin, supervised machine learning, thrombosis, anticoagulants, correlation of data

## Abstract

**Background:**

With the rapid development of artificial intelligence, prediction of warfarin dose via machine learning has received more and more attention. Since the dose prediction involve both linear and nonlinear problems, traditional machine learning algorithms are ineffective to solve such problems at one time.

**Objective:**

Based on the characteristics of clinical data of Chinese warfarin patients, an improved stacking ensemble learning can achieve higher prediction accuracy.

**Methods:**

Information of 641 patients from southern China who had reached a steady state on warfarin was collected, including demographic information, medical history, genotype, and co-medication status. The dataset was randomly divided into a training set (90%) and a test set (10%). The predictive capability is evaluated on a new test set generated by stacking ensemble learning. Additional factors associated with warfarin dose were discovered by feature selection methods.

**Results:**

A newly proposed heuristic-stacking ensemble learning performs better than traditional-stacking ensemble learning in key metrics such as accuracy of ideal dose (73.44%, 71.88%), mean absolute errors (0.11 mg/day, 0.13 mg/day), root mean square errors (0.18 mg/day, 0.20 mg/day) and R^2^ (0.87, 0.82).

**Conclusions:**

The developed heuristic-stacking ensemble learning can satisfactorily predict warfarin dose with high accuracy. A relationship between hypertension, a history of severe preoperative embolism, and warfarin dose is found, which provides a useful reference for the warfarin dose administration in the future.

## 1 Introduction

Warfarin is a medication used for anticoagulant therapy, primarily aimed at preventing and treating blood clot formation, such as deep vein thrombosis and pulmonary embolism. It is characterized by a narrow therapeutic range and significant dose variability. Despite these challenges, warfarin remains an effective option in managing thrombotic conditions. Research has found that important genetic factors for warfarin dosing are the variants in two genes, cytochrome P450 family 2 subfamily C member 9 (CYP2C9) and vitamin K epoxide reductase complex subunit 1 (VKORC1) (1–3). However, genetic testing is costly and warfarin dosing has wide inter- and intra-individual variability. In addition, when facing patients of the same race, a large number of genes are consistent, and factors other than genes need attention. In fact, patient clinical characteristics such as age, sex, weight, concomitant medications, and medical history affect warfarin dosing significantly (4). A study by Emery et al. (5) also showed that a patient's history of heart valve surgeries, namely, mitral valve replacement (MVR), aortic valve replacement (AVR), and tricuspid valve replacement (TVR), affects the level of anticoagulation targets. These surgeries can lead to changes in warfarin dosing, which were ignored in previous prediction studies.

On some occasions, clinicians rely on blood tests and their personal experience to determine the right dosage of warfarin for patients. However, incorrect dosing of warfarin can have severe and potentially fatal consequences. The use of personal experience as a determining factor is challenging to standardize and widely implement. Therefore, it is crucial to explore alternative methods, apart from blood tests, that can accurately prescribe the appropriate warfarin dose based on individual patient factors. This approach would not only reduce costs but also provide convenience to patients.

Warfarin stable dose prediction models based on machine learning for different racial populations have been developed (6–9). To improve prediction accuracy, people usually focus on comparing different algorithms and adjusting the hyperparameters. Some works ignore the impact of feature selection (10, 11). Although multiple linear regression algorithms have performed well in some dose prediction occasions, studies have shown that there may be a non-linear relationship between some factors and warfarin dose (12). In this case, linear regression is difficult to deal with such problems. To improve the accuracy and generalization ability of warfarin dose prediction, ensemble learning is gradually introduced (13–17).

In collaboration with medical centers in southern China, a comprehensive dataset was gathered, comprising information on 641 hospitalized patients who underwent either mechanical heart valve replacement (MHVR) or bioprosthetic heart valve replacement (BHVR). This dataset not only included clinical characteristics, concurrent medications, medical history, and surgical history but also incorporated data on the presence of severe embolic conditions before and after the surgeries. To handle multiple parameters effectively, feature selection methods were employed to identify the most relevant subset of features. Correlation coefficients were then utilized to assess the relationship between each variable and the dosage of warfarin, a commonly used medication. Building upon the optimal feature subset, a heuristic-stacking ensemble learning algorithm was developed to train a predictive model. This novel model integrated various feature selection methods and provided a comprehensive assessment of the impact of surgical history on warfarin dosage. The correlation coefficients were instrumental in determining the association between each variable and the appropriate warfarin dose. Evaluation metrics such as root mean square error (RMSE), coefficient of determination (*R*^2^), mean absolute error (MAE), and the proportion of patients within ±20% error were employed to gauge the accuracy of the predictions. The results demonstrated that the heuristic stacking ensemble learning approach outperformed other methods, delivering highly accurate warfarin dosage recommendations.

## 2 Material and methods

### 2.1 Datasets

The data of 641 warfarin patients from southern China were obtained through authorized patient medical records, including gender, age, weight, height, ethnicity, smoking and drinking status, surgical history, co-medication status, genotype, and personal medical history (see Table 1 for details). The mean age of the 641 patients was 46.6 years. The majority were Han Chinese (96.6%), and 259 of these 641 patients were men (40.4%). A majority (78.9%) had undergone mechanical heart valve replacement surgery and their international normalized ratio (INR) ranged from 1.7 to 2.5, while 21.1% underwent bioprosthetic heart valve replacement with an INR between 1.6 and 2.0. Warfarin is mainly used to treat thrombosis, which is an important risk factor for systemic thromboembolism and sudden death. In addition to the above two procedures, whether the patients had undergone thrombectomy or other information related to thrombosis was recorded. The results of Ambuj et al. (18) showed that patients with left atrial thrombosis should undergo thrombectomy promptly, and they concluded that atrial fibrillation causes left atrial thrombosis, so patients with atrial fibrillation (32.3%) were also included in the dataset. Several studies also found an association between warfarin usage and diabetes (19), coronary artery disease (20), and hypertension (21). There are 2.5%, 1.2%, and 7.6% of patients in these three conditions from our dataset, respectively.

**Table 1:** Demographic and clinical characteristics were collected from 641 patients.

Variables	All dataset (*N* = 641)	Training dataset (*N* = 577)	Test dataset (*N* = 64)	*p*-value
Basic characteristics				
Male	259 (40.4)	232 (40.2)	27 (42.2)	.80
Age (years)^a^	46.6 (10.9)	46.6 (10.9)	46.4 (11.1)	.88
Height (cm)^a^	160.6 (8.1)	160.6 (8.0)	160.8 (8.6)	.94
Weight (kg)^a^	56.4 (9.3)	56.3 (9.3)	57.4 (9.0)	.40
Han Chinese	619 (96.6)	558 (96.7)	61 (95.3)	1.00
Habit				
Smoking	67 (10.5)	61 (10.6)	6 (9.4)	.58
Drinking	33 (5.1)	30 (5.2)	3 (4.7)	1.00
Genetics				
CYP2C9 genotype				.44
*1/*1	582 (90.8)	529 (91.7)	53 (82.8)	
*1/*3	56 (8.7)	46 (8.0)	10 (15.6)	
*3/*3	3 (0.5)	2 (0.3)	1 (1.6)	
VKORC1 genotype				1.00
CC	6 (1.0)	5 (0.9)	1 (1.6)	
TC	122 (19.0)	109 (18.9)	13 (20.3)	
TT	513 (80.0)	463 (80.2)	50 (78.1)	
Surgery				
MHVR	506 (78.9)	453 (78.5)	53 (82.8)	.26
BHVR	135 (21.1)	124 (21.5)	11 (17.2)	.26
MVR	519 (81.0)	470 (81.5)	49 (76.6)	.27
AVR	287 (44.8)	255 (44.2)	32 (50.0)	.45
TVR	37 (6.8)	34 (5.9)	3 (4.7)	1.00
Thrombus removal	55 (8.6)	54 (9.4)	1 (1.6)	1.00
Comorbidity				
High blood pressure	49 (7.6)	44 (7.6)	5 (7.8)	1.00
Coronary heart disease	8 (1.2)	7 (1.2)	1 (1.6)	1.00
Diabetes	16 (2.5)	13 (2.3)	3 (4.7)	1.00
Atrial fibrillation	207 (32.3)	185 (32.1)	22 (34.4)	.79
Indication				
HSEBO	34 (5.3)	32 (5.5)	2 (3.1)	1.00
PSES	6 (0.9)	6 (1.0)	0 (0.0)	–
Medication				
Increase INR drug^b^	21 (3.3)	20 (3.5)	1 (1.6)	1.00
Decrease INR drug^b^	2 (0.3)	2 (0.3)	0 (0.0)	–
Amiodarone	9 (1.4)	8 (1.3)	1 (1.6)	–
Tartine	8 (1.2)	8 (1.3)	0 (0.0)	–
Inducer^c^	2 (0.3)	2 (0.3)	0 (0.0)	–
Thyroxine tablets	2 (0.3)	2 (0.3)	0 (0.0)	–
Fluconazole	3 (0.5)	3 (0.5)	0 (0.0)	–
Aspirin	8 (1.2)	7 (1.2)	1 (1.6)	–

HSEBO, history of severe embolism before operation; PSES, postoperative severe embolism symptoms.

^a^ Data was shown as mean (standard deviation), and others were shown as frequency (%).

^b^ All other drugs that may affect warfarin dose except for Amiodarone, Tartine, Inducer, Thyroxine Tablets, Fluconazole, and Aspirin.

^c^ CYP2C9 and VKORC1 inducers.

Since patients' genotypes are unpredictable, they lead to a large difference in the amount of data between the common CYP2C9*1/*1 (90.8%) and the rare CYP2C9*3/*3 (0.5%). This problem is also present in the VKORC1 genotype case. VKORC1 has three manifestations CC, TC, and TT accounting for 1.0%, 19.0%, and 80.0%, respectively. This extreme data distribution will cause data leakage potentially, thus allowing the model to perform high prediction falsely. Because of this, data preprocessing is particularly important (preprocessing results are shown in Supplementary Table S1). *χ*^2^ test (for continuous variables) and a Fisher exact test (for categorical variables) were used to verify both the training set (90%) and test set (10%). We found that “Severe postoperative plagiocephaly”, “Decrease INR drug”, “Increase INR drug”, “Amiodarone”, “Tartine”, “ Inducer”, “Thyroxine Tablets”, “Fluconazole”, and “Aspirin”, these eight variables, should not be included in the subsequent machine learning model training to prevent data leakage due to insufficient data for Fisher's exact test. Although the co-administration data described above are scarce, it has been shown (16) that co-administration can have an effect on warfarin dose, so it should be included in the study for correlation analysis.

The classification of patients into high (>4 mg/day), moderate (>2 mg/day and <4 mg/day), and low (<2 mg/day) dose levels (22) revealed many missing data, mainly focusing on co-medication, smoking status, drinking status, medical history, and genotype of patients at high and low doses. Faced with these data deficiencies, it is necessary to find the optimal subset of features. This study uses recursive feature elimination with cross-validation (RFECV), correlation coefficient-based filtering method, and gini importance-based bidirectional search (BDS) (23, 24) to find the globally optimal feature subset.

### 2.2 Methods

The authors conducted correlation tests based on these data. The tests aimed to indicate the degree of correlation between the targeted characteristic variables. The correlation coefficient can be used to objectively represent the correlation. However, some correlation coefficients can only indicate whether there is a linear relationship or not. So Pearson's coefficient (25), Spearman's coefficient (26), and Kendall's tau-b coefficient tests (27) were introduced to comprehensively describe the relationship. The arithmetic mean of the absolute values of the three coefficients was analyzed to find out the correlation between different variables and warfarin dose. In this study, IBM SPSS 26.0 statistical software was used to calculate the three correlation coefficients, and the results are shown in Supplementary Figures S1–S4. Three correlation coefficients showed that the CYP2C9 and VKORC1 genotypes of patients had a high correlation with warfarin dose, and patients with CYP2C9 *3 mutation required less warfarin dose (*p* < 0.001). VKORC1-1173 locus was a CT mutation and CC was a wild type. Patients with CT mutation required less warfarin dose than wild type, and patients with TT mutation required less dose than CT mutation (*p* < 0.001), which is consistent with the previous findings (28).

**Figure 1:**
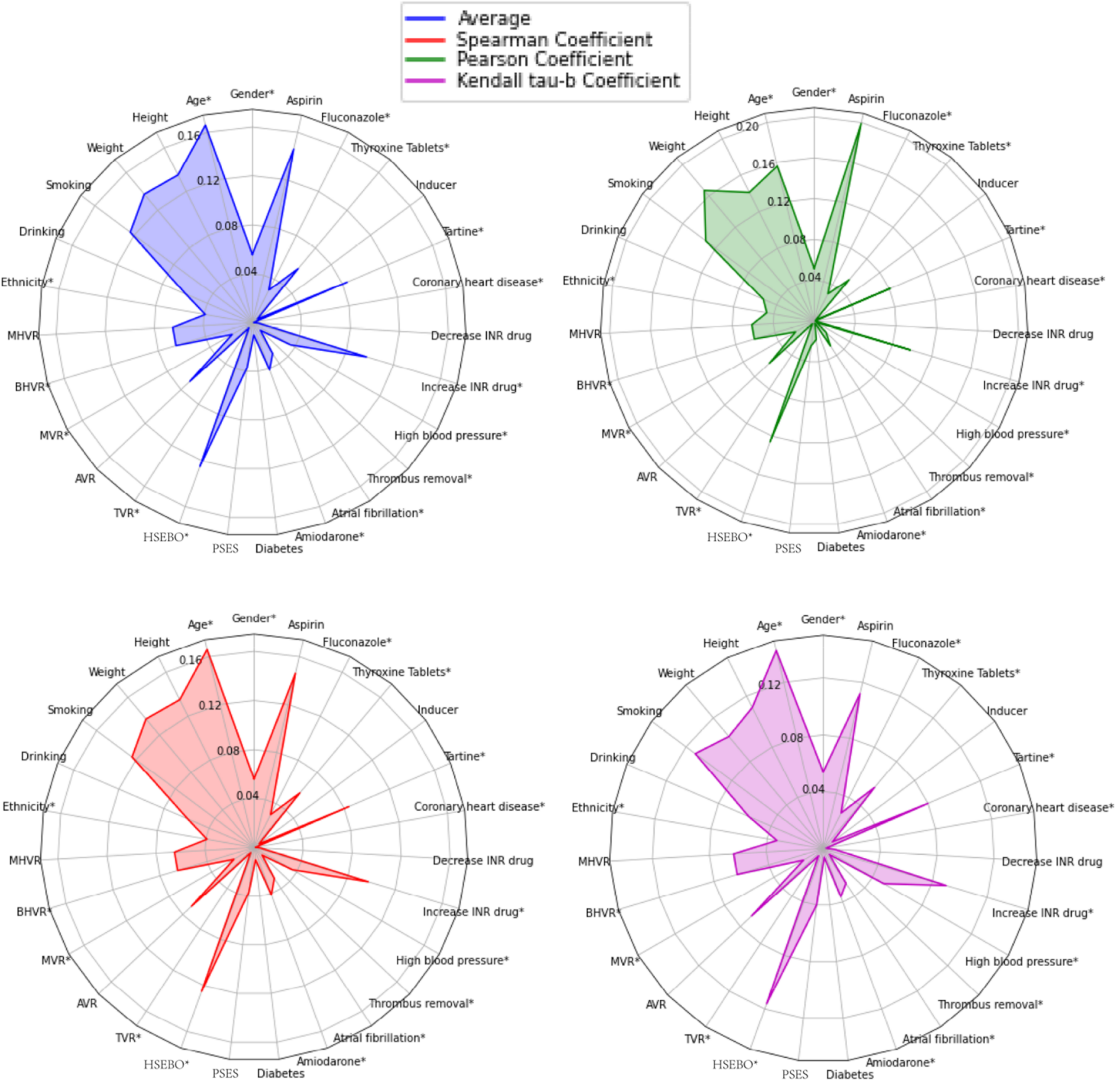
Descriptions of spearman coefficient, Pearson coefficient, and Kendall Tau-B coefficient, as well as the arithmetic mean of the above three for the 27 variables correlated with warfarin dose. *Negatively correlated with warfarin dose. HSEBO, history of severe embolism before operation; PSES, postoperative severe embolism symptoms.

In addition, to find out other influences with weaker correlations, we temporarily removed CYP2C9 and VKORC1 genotypes and found that the four variables of patients' age, height, weight, and whether they were taking Aspirin had higher correlations compared with others, as shown in Figure 1. Smokers require higher warfarin doses than non-smokers (*p* < 0.001). Some studies (29) have also shown that tobacco increases warfarin clearance, which leads to a decrease in warfarin action, so clinicians should monitor patients' smoking status closely. If the combination of Aspirin with warfarin may increase the risk of bleeding, the risk of thromboembolism and bleeding should be dynamically evaluated, and INR and bleeding should be monitored intensively (*p* < 0.001). In the Tartine class, fluvastatin and simvastatin lead to an enhanced warfarin action, and rosuvastatin may enhance warfarin action but with large individual variation. Patients who require a combination of Tartine class drugs and warfarin should be monitored after initiation of Tartine class drugs and throughout the whole treatment period (*p* < 0.05). One study (30) showed that patients who underwent AVR surgery required more warfarin (*p* < 0.05), and there was also a linear relationship between the five medical histories on warfarin dose, with a negative correlation between HSEBO and warfarin dose (*p* < 0.001). The possible association between variables, exhibited a low correlation with warfarin, and on the other hand, insufficient data for certain variables could lead to non-significant statistically. The effect of variables on warfarin dose should also be considered in conjunction with the subsequent search for a global optimal subset of features, and the correlation coefficients were calculated for subsequent feature selection.

The correlation analysis showed that the six variables of patients, CYP2C9 genotype, VKORC1 genotype, age, height, weight, and Aspirin, have a large effect on warfarin dose, which is agreed to by Liyan et al. (31). The distribution of training data affects the accuracy of model prediction, so the above variables were screened out to observe their distribution in the dataset as shown in Supplementary Figure S5.

## 3 Results and discussion

### 3.1 Optimal feature subset

**Figure 2:**
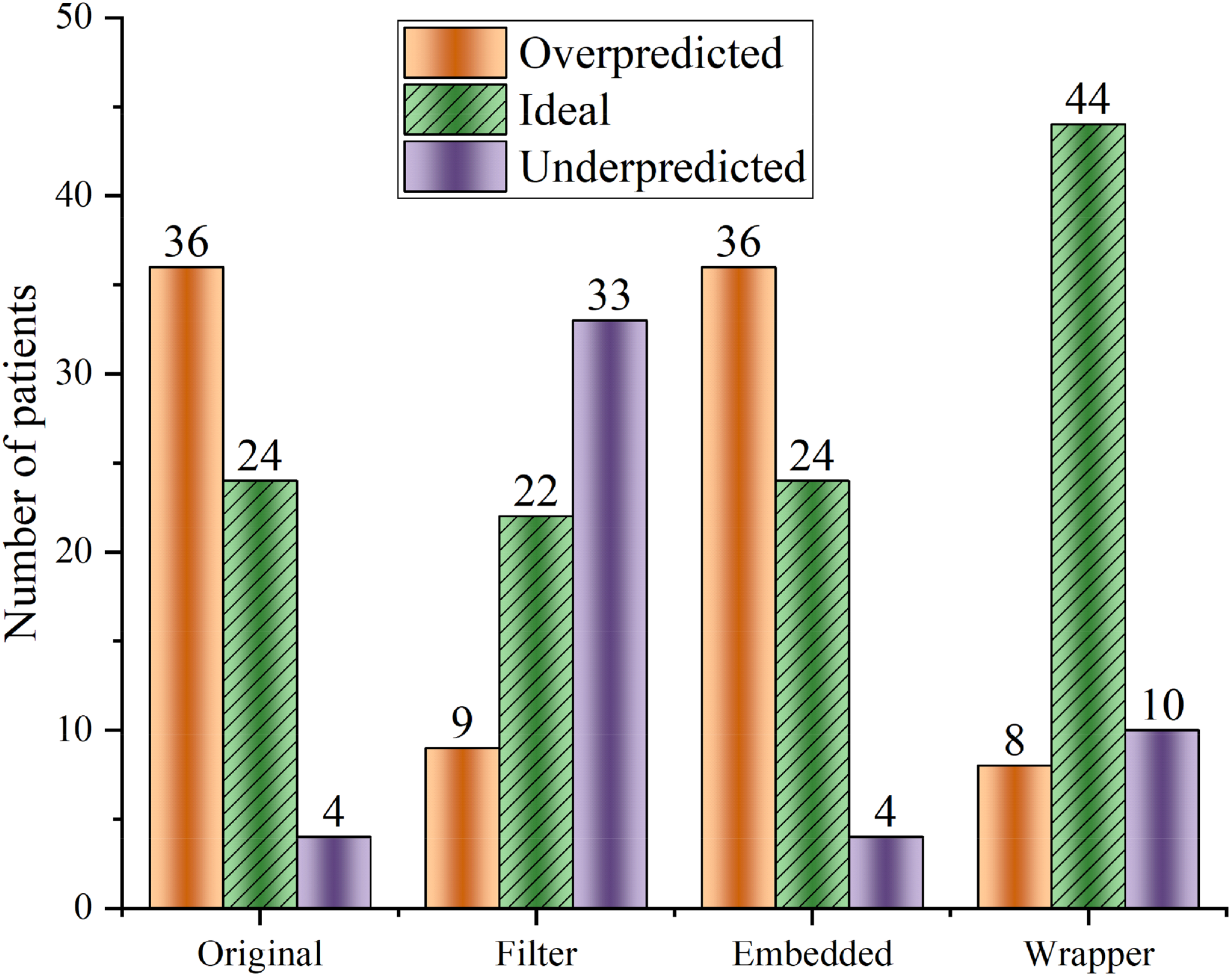
Random forest regression was used to predict warfarin dose based on data optimized by three feature selection methods. “Ideal” refers to the warfarin dose prediction error within ±20%. “Original” refers to the dataset that has not undergone feature selection.

Feature selection is very suitable for these cases with high dimensionality and complex relationships. Feature selection can determine the upper limit of the model's accuracy, and the algorithm selection only allows the model to converge to this limit infinitely. Random forest regression (RF) is used to test the effectiveness of the three feature selection methods. Figure 2 shows that the Wrapper method works best (ideal cases are more than others), and the extrapolation capability is substantially improved compared to the other two feature selection methods.

Some studies (32) demonstrated that bidirectional feature search methods can effectively avoid getting trapped in a local optimization point. The global optimal subset of features after screening based on the BDS method contains nine variables: age, smoking history, VKORC1, CYP2C9, MHVR, BHVR, blood pressure, coronary heart disease, and HSEBO. Feature importance was calculated using scikit-learn (33), and the results are shown in Supplementary Figure S6. Wypasek's study (30) showed an effect of coronary artery disease on warfarin dose, which is necessary to include as a variable.

### 3.2 Performance measures

Four indicators, *R*^2^, RMSE, MAE, and the proportion of patients with prediction errors within ±20%, are used to evaluate the prediction accuracy of the algorithms, which are calculated below (13, 22):

(1) $$\mathrm{MAE}=\frac{1}{n}\sum_{i=1}^{n}|{\hat{y}}_{i}-y_{i}|$$

(2) $$\mathrm{RMSE}=\sqrt{\frac{1}{n}}\sum_{i=1}^{n}{\left({\hat{y}}_{i}-y_{i}\right)}^{2}$$

(3) $$R^{2}=1-\frac{\sum_{i=1}^{n}{\left(y_{i}-{\hat{y}}_{i}\right)}^{2}}{\sum_{i=1}^{n}{\left(y_{i}-\bar{y}\right)}^{2}}$$

(4) $$\mathrm{ER}R_{\pm 20\text{\%}}=\frac{D_{ideal}}{D_{test}}\;\times \;100\text{\%}$$

where $y_{i}$ is the actual output, $\bar{y}$ is the sample mean value, *n* and $D_{test}$ are the number of samples in the test set, ${\hat{y}}_{i}$ is the predicted output, and $D_{ideal}$ is the number of samples with an error within ±20% between predicted and actual output.

After literature research, it was found that RF (8), GBRT (17), MLR (34), and SVR (8) performed well in warfarin dose prediction, and RF and GBRT are one of the commonly used ensemble learning algorithms for Bagging and Boosting, respectively. Stacking ensemble learning is an algorithm that can easily fall into overfitting. So in addition to introducing K-fold cross-validation, KRR, SGD, Bayes, and GPR were selected as base learners, where KRR and SGD introduced L2 parametric regularization methods to reduce overfitting, and Bayes used Gaussian prior probability distribution instead of regularization methods to reduce overfitting (35). GPR was chosen because it uses the kernel trick in KRR to implement a Gaussian process. GPR's predictions are interpolations of observations and its extrapolation is weak (36), but an algorithm with different features is needed in stacking ensemble learning. To control the time cost, the subsequent selection of the base learner does not consider artificial neural networks.

Table 2 summarizes the preliminary results of the above four indicators. GPR and SVR are the two most effective algorithms; this good prediction accuracy result cannot be attributed to the algorithms only but illustrates the importance of the global optimal feature subset (Figure 2). On the other hand, considering only *R*^2^, RMSE, and MAE (Equations 1–3), these three indicators, one of Bayes and MLR should be chosen as the meta-learner. However, these two algorithms perform poorly at ERR _± 20%_ (Equation 4), so SVR is more suitable as a meta-learner. The kernel function of SVR uses radial basis functions. All other parameters use the default parameters from scikit-learn.

**Table 2:** Comparison of prediction performance of machine learning algorithms.

Algorithms	*R* ^2^	RMSE	MAE	ERR _± 20%_
RF	0.41	0.79	0.57	68.75%
GPR	0.50	0.72	0.56	68.75%
SGD	0.50	0.73	0.56	64.06%
GBRT	0.46	0.76	0.55	64.06%
Bayes	0.50	0.72	0.56	64.06%
KRR	0.41	0.79	0.60	59.38%
MLR	0.50	0.72	0.56	60.94%
SVR	0.45	0.76	0.55	70.31%

### 3.3 Heuristic-stacking ensemble learning

The commonly used methods for ensemble learning are bagging, boosting, and stacking. However, the first two are often used in classification tasks (37). In addition, both methods are homogeneous ensemble learning, while stacking is heterogeneous ensemble learning, which can effectively integrate different algorithms to solve the problem of simultaneous non-linearity and linearity. Stacking ensemble learning should satisfy the following conditions as much as possible (38):

• The prediction accuracy of the meta-learners should be better than others.
• Selected algorithms should have different characteristics.

Traditional-stacking ensemble learning has obvious drawbacks, as shown in Supplementary Figure S7. Traditional-stacking ensemble learning uses arithmetic averaging to generate a new test set. This method will erase the superiority of the base learner in the training set. Wolpert said the improvement direction of stacking ensemble learning can be considered with RMSE (Equation 2) reciprocal weighting and multilayer grid structure (38). But this grid structure suffers from serious overfitting and therefore is not included in the subsequent improvement directions. Weighted-stacking ensemble learning is a weighted average of the inverse of the RMSE instead of the arithmetic average, as shown in Supplementary Figure S8. However, there is no objective metric to measure the difference between the two algorithms. Bidirectional feature search is recruited to choose an algorithm (meta-learner) for stacking. The pseudocode of heuristic-stacking ensemble learning is shown in Supplementary Table S2. There are three requirements to use proposed heuristic-stacking ensemble learning:

• Base learners added by forward learner search cannot be deleted by backward learner search.
• Base learners deleted by backward learner search cannot be added by forward learner search.
• Stop searching when both searches find the same combination of base learners.

**Figure 3:**
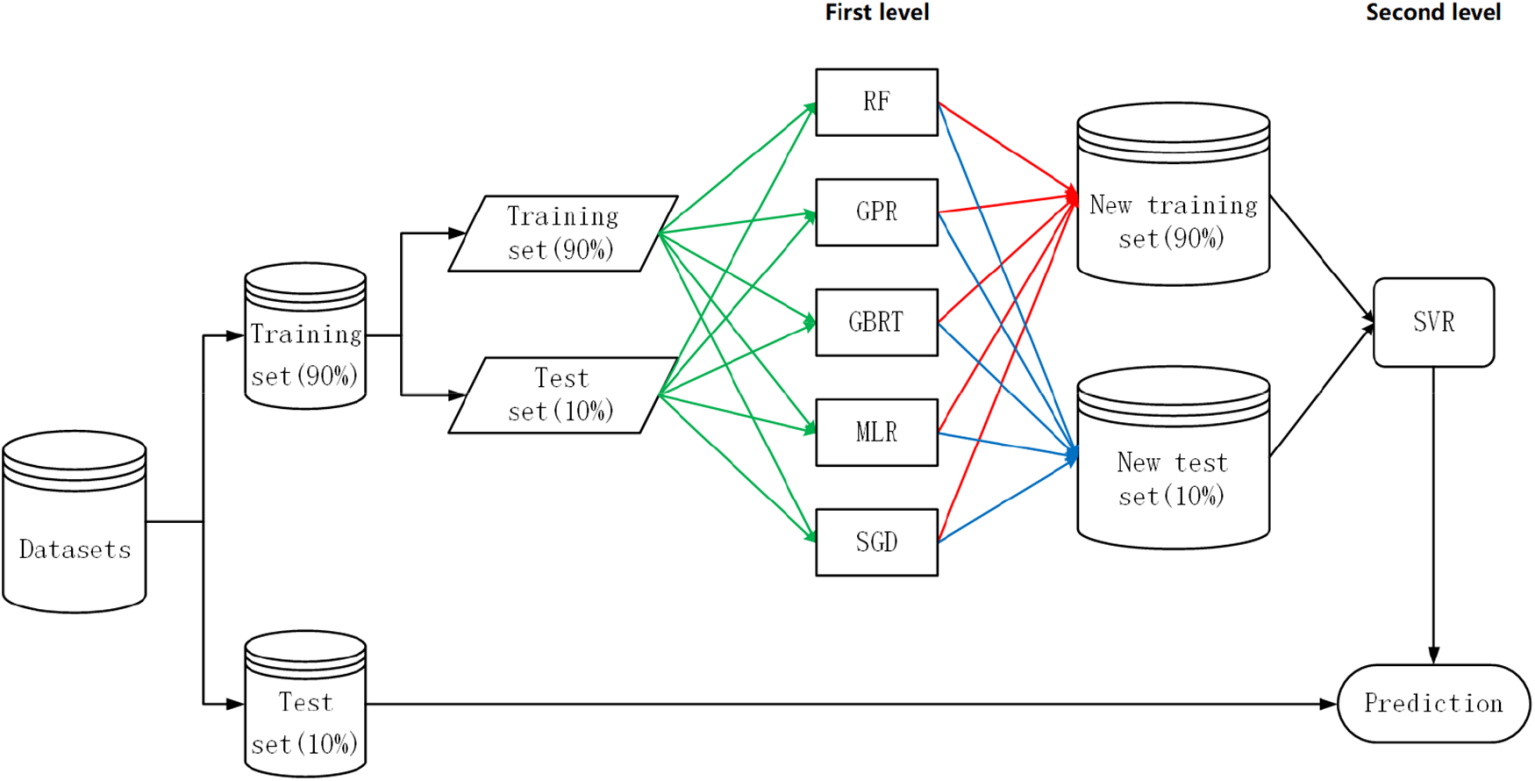
Heuristic-stacking ensemble learning development strategy. RF, random forest; GPR, Gaussian process regression; GBRT, gradient boost regression tree; SGD, stochastic gradient descent; MLR, multiple linear regression; SVR, support vector regression. The green line indicates that the training set and test set are stacked to form a new training set and new test set. The red lines indicate that the training set is sent to each algorithm for 5-fold cross-validation before prediction processing. The blue line indicates that each algorithm was an ensemble for the test set after taking a weighted average based on the reciprocal of the RMSE.

Our proposed specific strategy for heuristic-stacking ensemble learning is shown in Figure 3, and the combination of five algorithms (RF, GPR, GBRT, MLR, and SGD) is the result of a bidirectional search. Because the deviation of the dataset generated by the base learner from the original dataset does not accurately represent the proportion of patients with a prediction error within ±20% (Equation 4), 10% of the data was left as an additional test set at the beginning to calculate the proportion of patients with a prediction error within ±20% (Equation 4).

To quantify performance differences, three ensemble algorithms are compared: traditional-stacking ensemble learning, weighted-stacking ensemble learning (using RMSE reciprocal weighting process), and proposed heuristic-stacking ensemble learning. The stacking ensemble learning algorithms use scikit-learn (33) to build the prediction models and run them in Python 3.7.2. The predicted performances are shown in Table 3.

**Table 3:** Comparison of three stacking ensemble learning prediction performances.

Algorithms	*R* ^2^	RMSE	MAE	ERR _± 20%_
Traditional-stacking	0.82	0.20	0.13	71.88%
Weighted-stacking	0.83	0.20	0.13	71.88%
Heuristic-stacking	0.87	0.18	0.11	73.44%

### 3.4 Discussion

One can see that the heuristic-stacking ensemble learning is better than the other two learnings, even though the other two recruit more base learners (see Supplementary Figures S7, S8). ERR _±20%_ (Equation 4) of heuristic-stacking ensemble learning can reach 73.44%.

Warfarin dose is prone to extreme bias in the collection of clinical data for a particular variable due to the complexity and variety of influencing factors. The high cost of data collection leads to insufficient data volume, which in turn creates the problem of data leakage. If the prediction model has known data characteristics of a variable, prediction results will lead researchers to mistakenly believe that adding some variables into a model will effectively improve the accuracy. Therefore, when dividing the training and test sets, it is necessary to consider whether there is a connection between these two groups. A statistical analysis is needed such as a test of variance between the two sets unless there is enough data.

The BDS feature selection method gives the global optimal feature subset. The improvement of model prediction accuracy by this method is better than the RFECV and filtering methods. Furthermore, the global optimal feature subset indicates that hypertension and a history of severe preoperative embolism are important for predicting warfarin doses. Clinicians should pay more attention to patients with these two situations in the future.

Moreover, there is a lack of variables like genotype that have a strong influence on warfarin dose. An evaluation index fitting the current situation is needed until a strong influence is found. For example, the International Warfarin Pharmacogenome Consortium (IWPC) (22) proposed that within ±20% error counts as a clinically meaningful evaluation metric.

Future studies could complement warfarin studies with prospective data and experiments. For example, the experience of doctors can be used as the results of the control group. The results of the experimental group are given by the machine learning model. When the results given by machine learning deviate significantly from the doctor's experience and the patient's response after taking the corresponding dose is different, this time period can be set as the follow-up period. Then, the machine learning model needs to be adjusted and intervened.

## 4 Conclusion

The prediction performance of the heuristic-stacking ensemble learning proposed in this study is better than traditional ones, with a proportion of patients within ±20% error of the prediction reaching 73.44% and the degree of regression fit *R*^2^ reaching 0.87. The BDS feature selection method is significantly better than the RFECV and filtering methods to select the suitable meta-learners. When using ensemble learning in warfarin dose prediction, to be alert to data leakage when data volume is insufficient, feature selection can play a great role to solve this problem. In addition, our results suggest that clinicians should pay attention to hypertension and a history of severe preoperative embolism. They can collect data from these aspects when determining a patient's stable warfarin dose to develop new evaluation metrics in the future.

## Data Availability

Data is not available due to legal restrictions. Please contact the corresponding author(s) for further enquiries.
