# Supplementary material for: Improved stacking ensemble learning based on feature selection to accurately predict warfarin dose
Source: Front Cardiovasc Med. 2024 Jan 19;10:1320938. doi: 10.3389/fcvm.2023.1320938 (PMC10834785; doi:10.3389/fcvm.2023.1320938)
Supplement: Supplementary file 1 [file Datasheet1.pdf]

Supplemental material

Improved stacking ensemble learning based on feature selection to accurately predict warfarin dose

Mingyuan Wang[2, §], Yi-yi Qian[1, §, *], Yaodong Yang[2, **], Haobin Chen[3], Wei-Feng Rao[2]

[1] Department of Pharmacy, Fuwai Yunnan Cardiovascular Hospital, Kunming, 650000, P. R. China

[2] School of Mechanical Engineering (Shandong Institute of Mechanical Design and Research), Qilu University of Technology (Shandong Academy of Sciences), Jinan, Shandong, 250353, P. R. China

[3] Department of Pathology, Qujing First People's Hospital, Qu Jing, Yunnan, 655000, P. R. China

[§]These authors contributed equally to this work and should be considered co-first authors.

Corresponding Authors

* E-mail address: 1270117602@qq.com

**E-mail address: yaodongy@qlu.edu.cn

Table S1 Details of data description and data pre-processing.

No.	Feature Name	Description	Data preprocessing (if any)
1	Age (year)	Continuous variable	
2	Height (cm)	Continuous variable	
3	Weight (kg)	Continuous variable	
4	Gender	0: Male; 1: Female	
5	Ethnicity	0: No; 1: Yes	1 if Han Chinese and 0 if Chinese minority
6	Smoking	0: No; 1: Yes	
7	Drinking	0: No; 1: Yes	
8	Thrombus removal	0: No; 1: Yes	
9	MHVR	0: No; 1: Yes	
10	BHVR	0: No; 1: Yes	
11	MVR	0: No; 1: Yes	
12	AVR	0: No; 1: Yes	
13	TVR	0: No; 1: Yes	
14	VKORC1 genotype	0: CC (Wildtype)<br>1: TC (Heterozygous mutant)<br>2: TT (Homozygous mutant)	
15	CYP2C9 genotype	0: Wildtype (*1/*1)<br>1: 1 Loss of function (*1/*3)<br>2: 2 Loss of function (*3/*3)	
16	High blood pressure	0: No; 1: Yes	
17	Coronary heart disease	0: No; 1: Yes	
18	Diabetes	0: No; 1: Yes	
19	Atrial fibrillation	0: No; 1: Yes	
20	HSEBO	0: No; 1: Yes	
21	PSES	0: No; 1: Yes	
22	Increase INR drug[a]	0: No; 1: Yes	1 if taking Increase INR drug else 0
23	Decrease INR drug[a]	0: No; 1: Yes	1 if taking Decrease INR drug else 0
24	Amiodarone	0: No; 1: Yes	1 if taking Amiodarone else 0
25	Tartine	0: No; 1: Yes	1 if taking Tartine else 0
26	Inducer[b]	0: No; 1: Yes	1 if taking Inducer else 0
27	Thyroxine Tablets	0: No; 1: Yes	1 if taking Thyroxine Tablets else 0
28	Fluconazole	0: No; 1: Yes	1 if taking Fluconazole else 0
29	Aspirin	0: No; 1: Yes	1 if taking Aspirin else 0
30	Stable warfarin dose (mg/day)	Output variable	

Abbreviations: HSEBO, history of severe embolism before operation; PSES, postoperative severe embolism symptoms.

[a]All other drugs that may affect warfarin dose except for Amiodarone, Tartine, Inducer, Thyroxine Tablets, Fluconazole, and Aspirin.

[b]CYP2C9 and VKORC1 inducers.

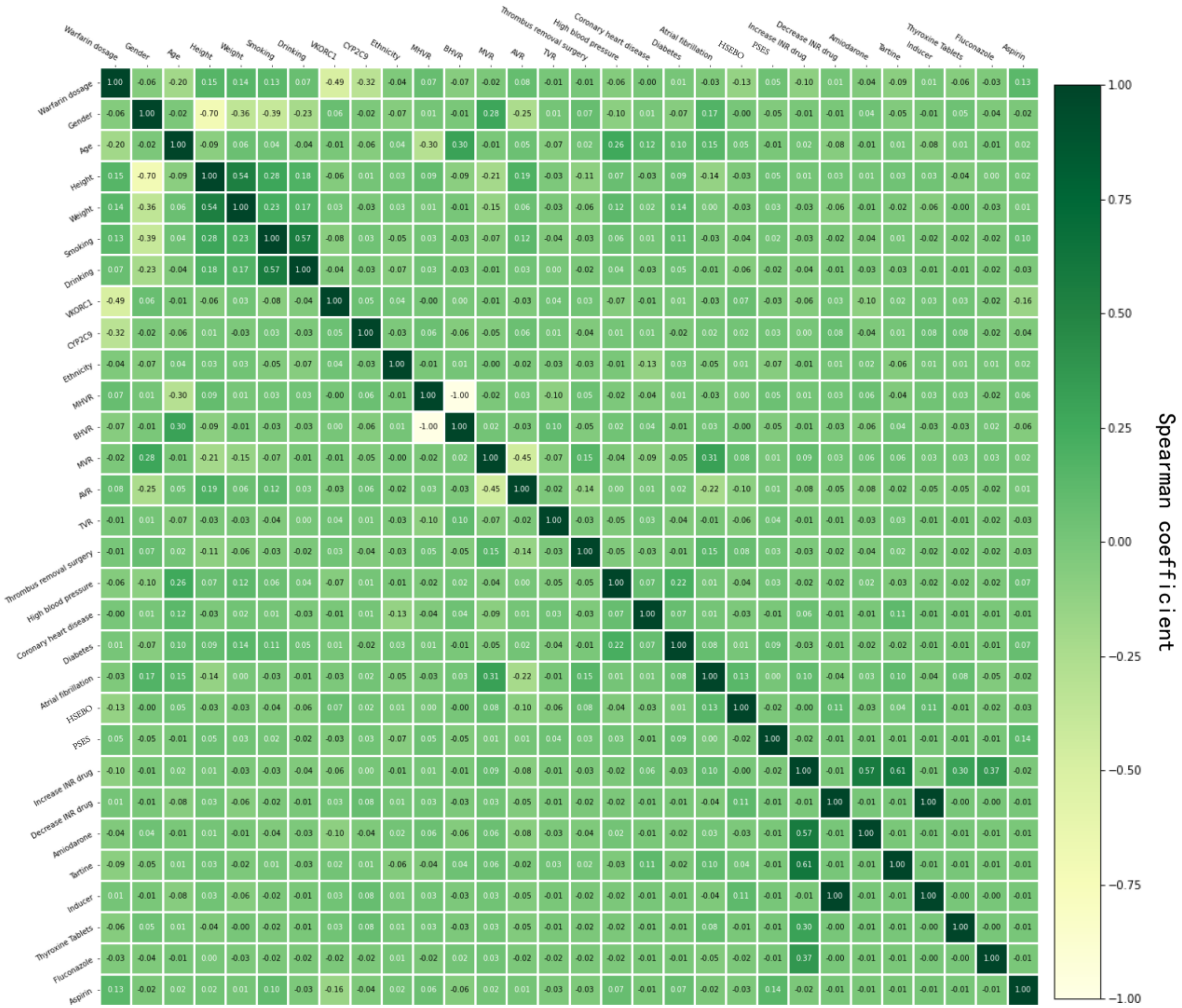

Fig. S1. Heat map of Spearman coefficient.

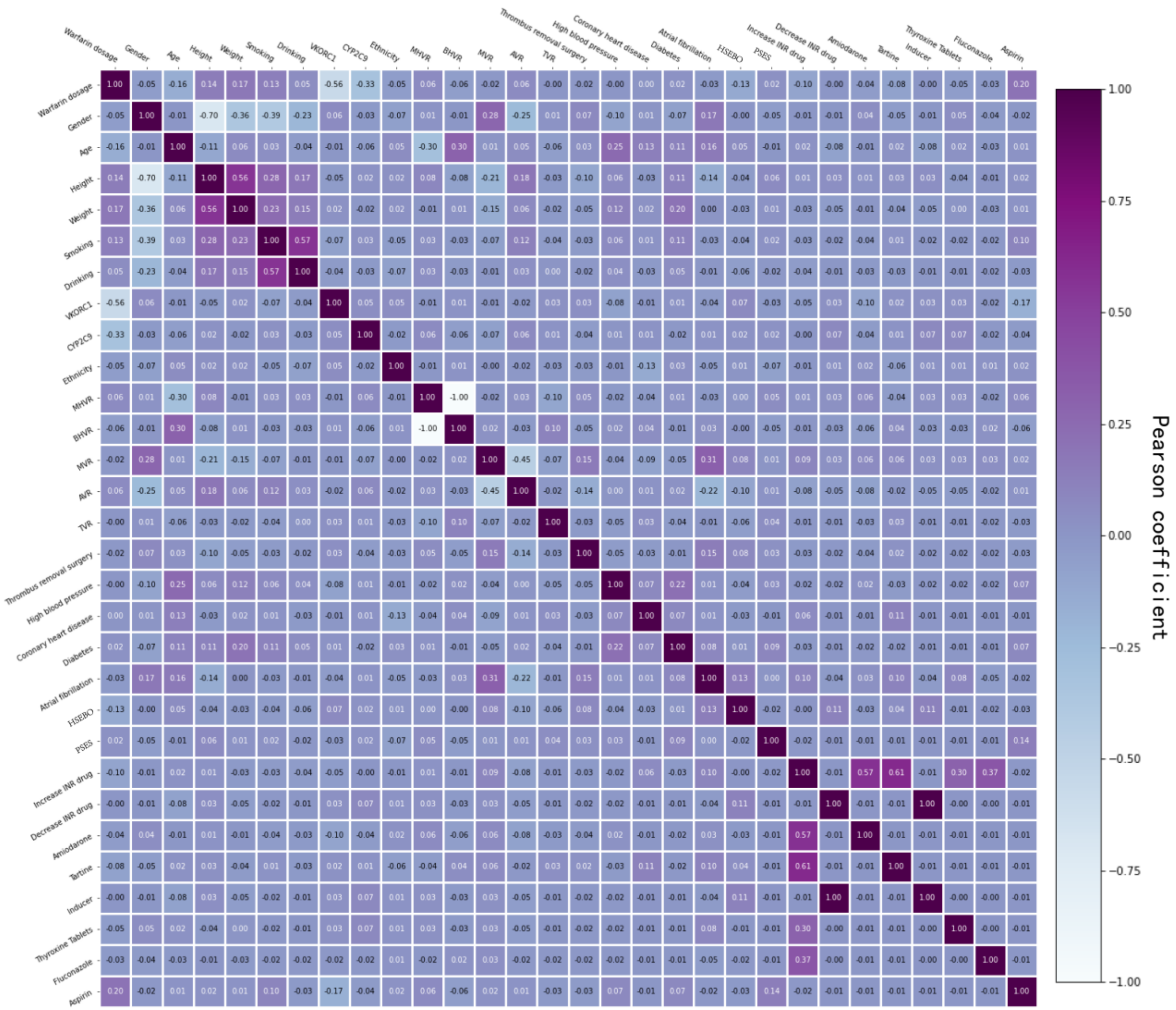

Fig. S2. Heat map of Pearson coefficient.

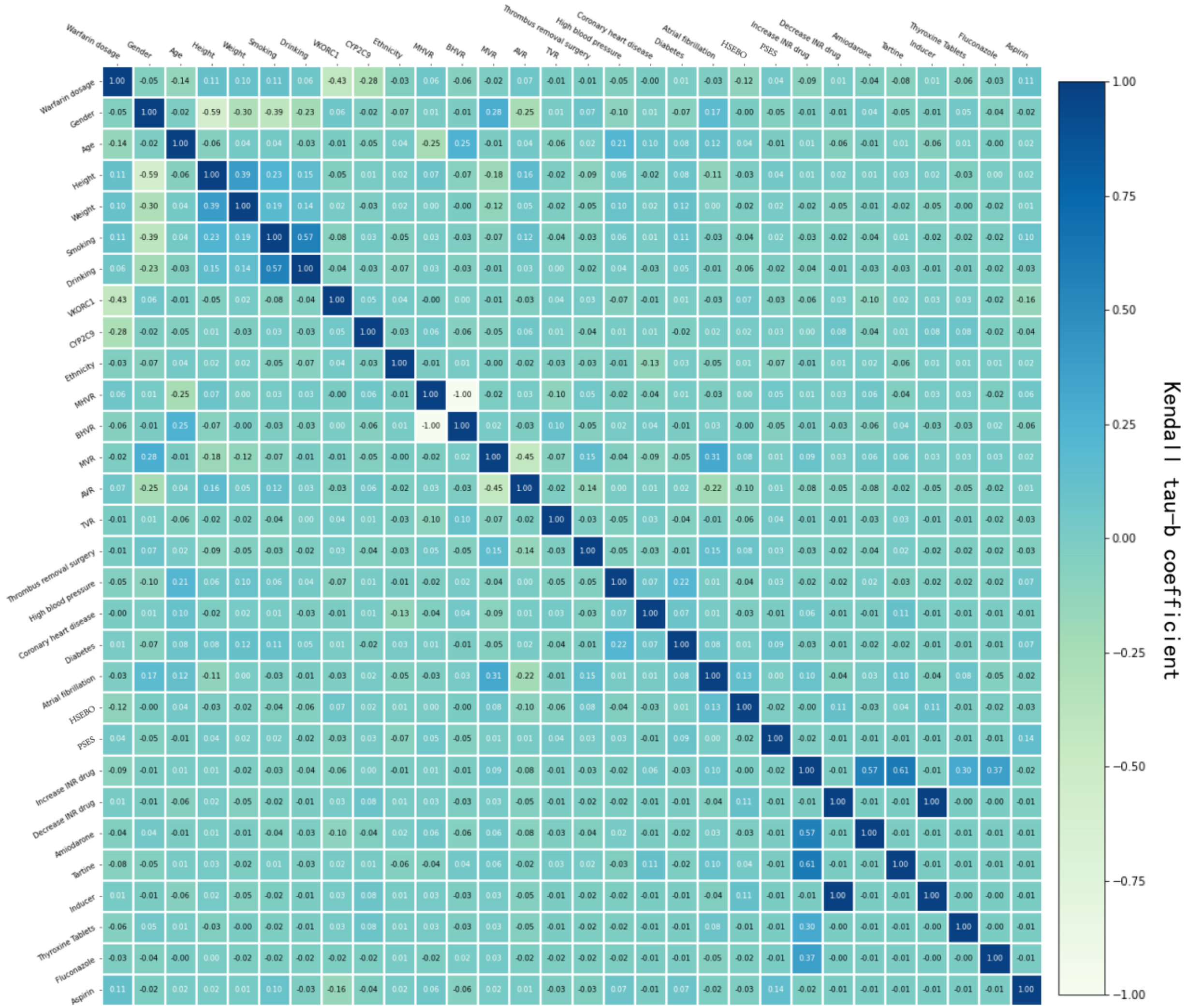

Fig. S3. Heat map of Kendall tau-b coefficient.

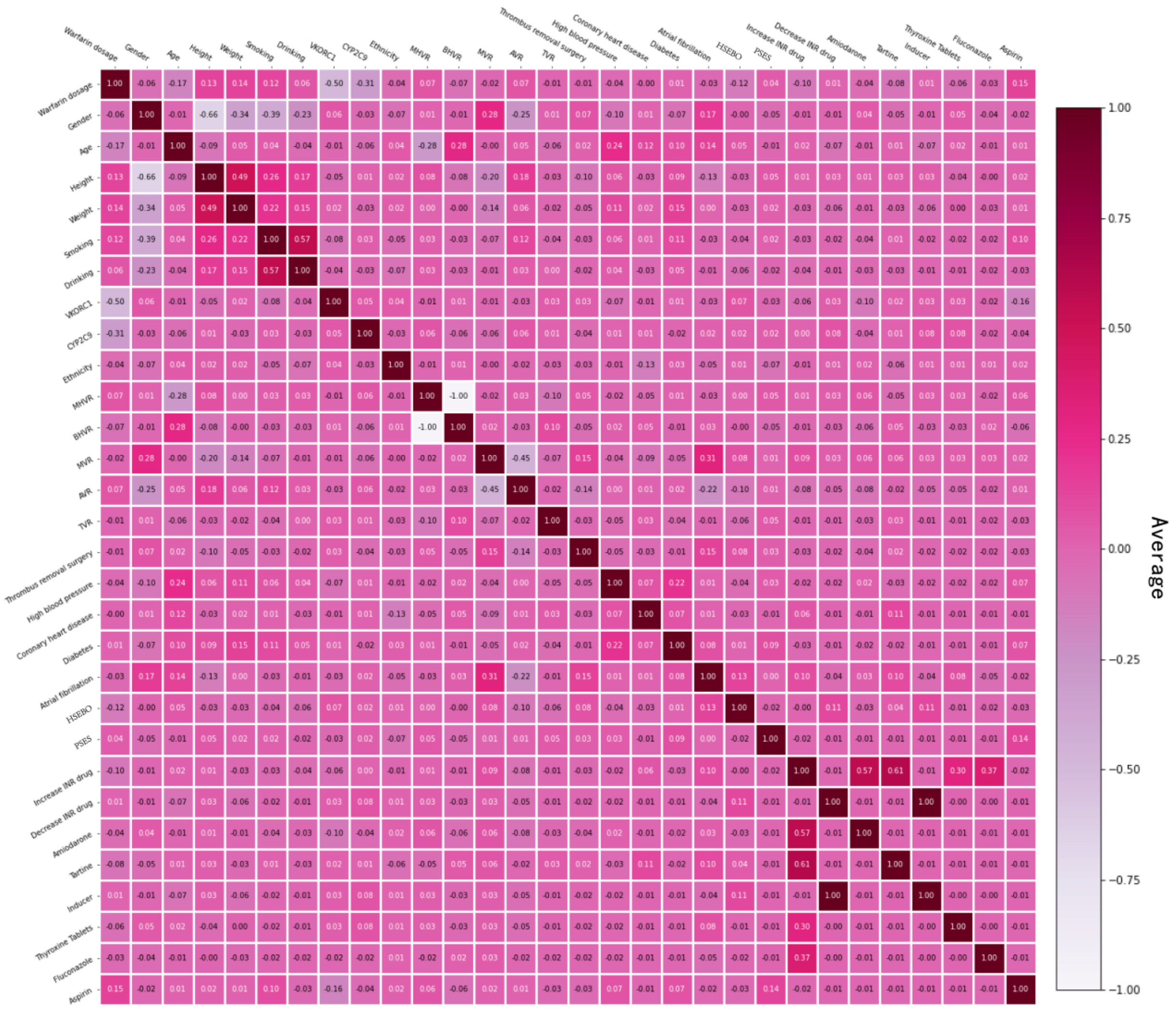

Fig. S4. Heat map of arithmetic mean of Spearman coefficient, Pearson coefficient, and Kendall tau-b coefficients.

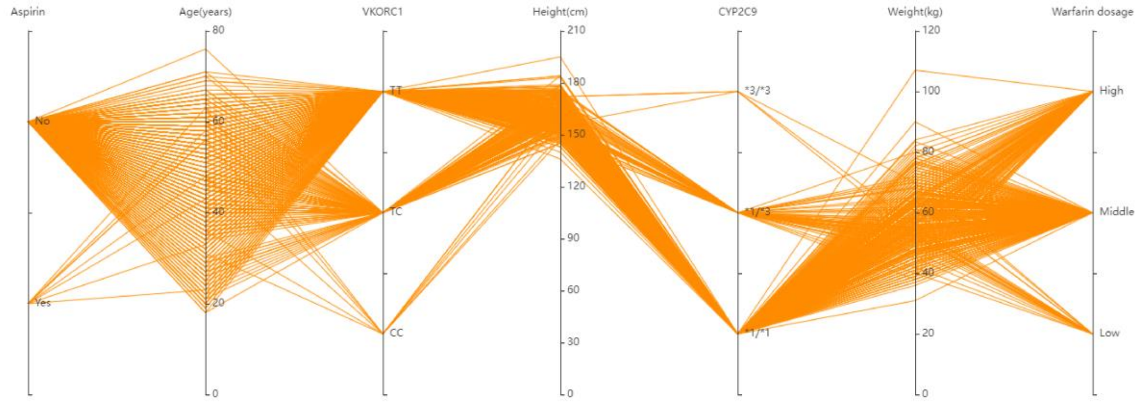

Fig. S5. Parallel axis plots of 6 highly correlated variables.

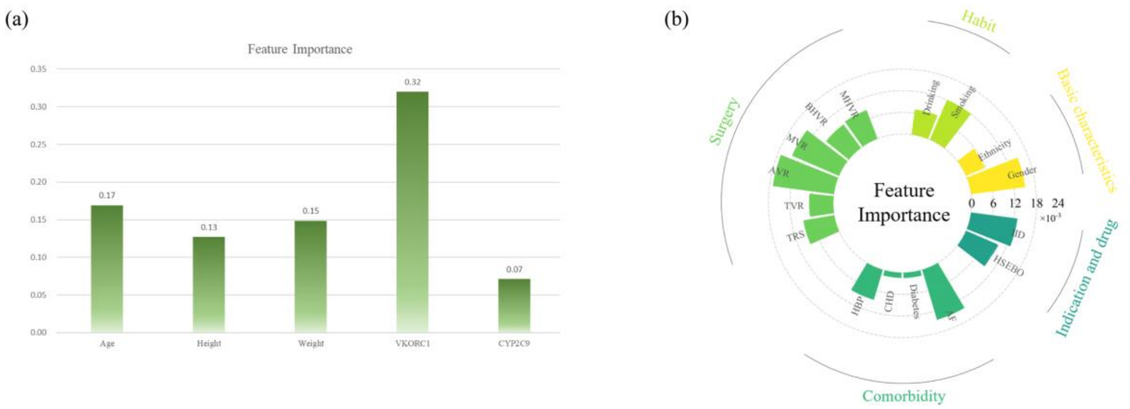

Fig. S6. Feature importance calculation results of the 21 variables involved in model training: (a) top five significant/important variables; (b) the remaining variables.

Abbreviations: HSEBO, history of severe embolism before operation; TRS, thrombus removal surgery; HBP, high blood pressure; CHD, coronary heart disease; AF, atrial fibrillation; IID, increase INR drug.

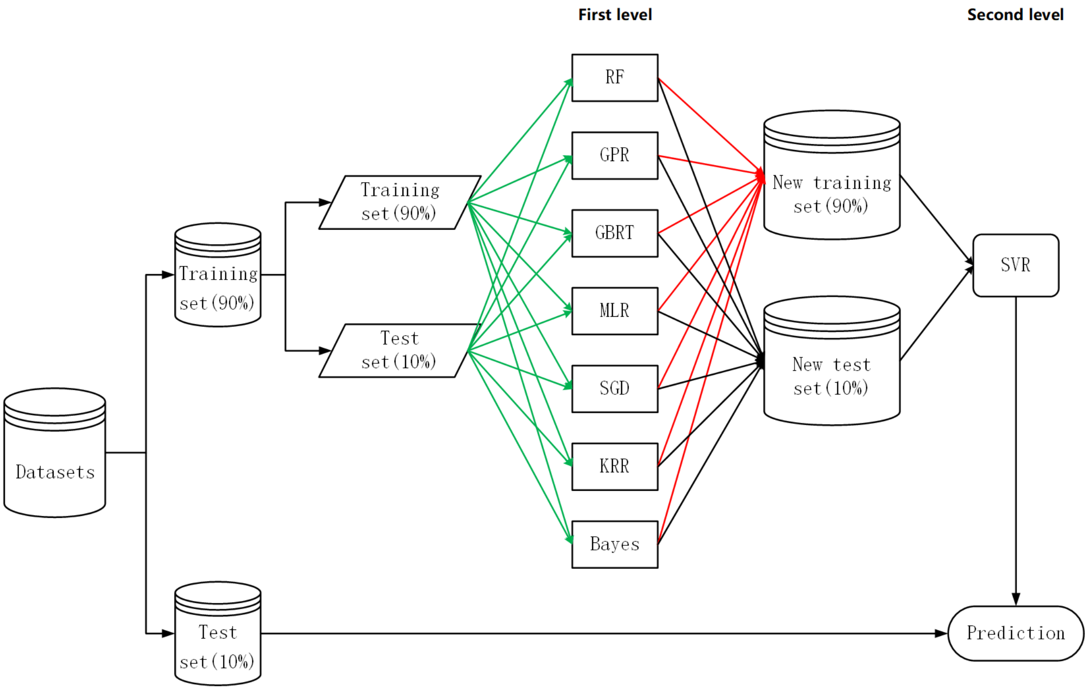

Fig. S7. Traditional-stacking ensemble learning strategy.

Note: The green line indicates that the training set and test set are stacked to form a new training set and new test set. The red lines indicate that the training set is sent to each algorithm for 5-fold cross-validation. The black line indicates that each algorithm was integrated for the test set after taking an arithmetic average.

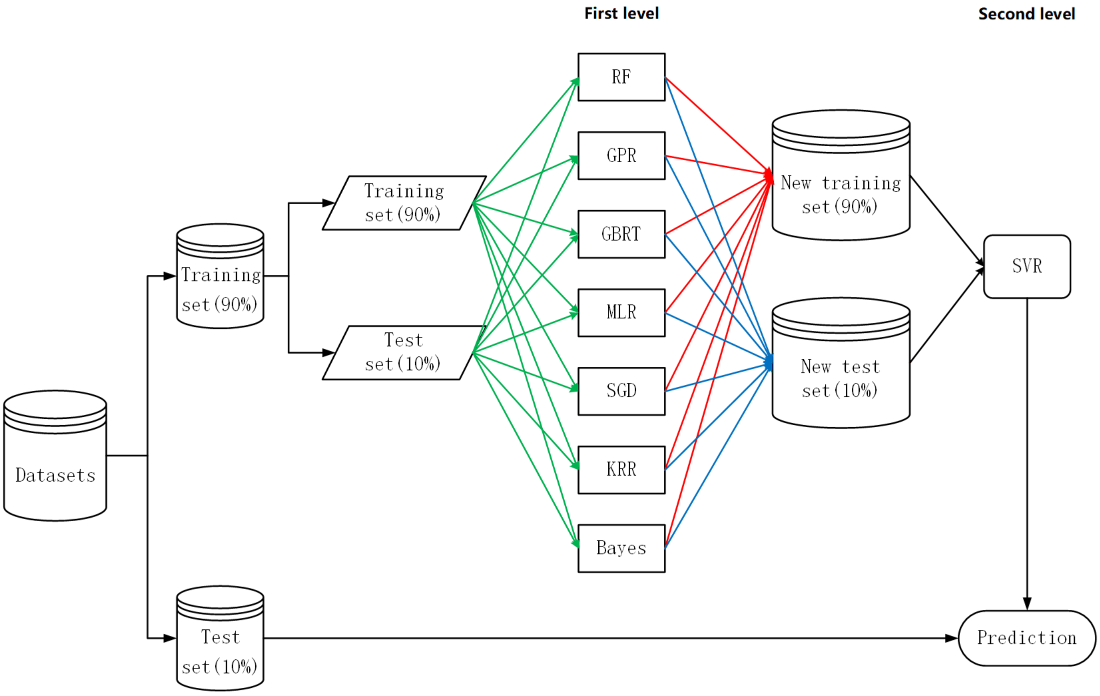

Fig. S8. Weighted-stacking ensemble learning strategy.

Note: The green line indicates that the training set and test set are stacked to form a new training set and new test set. The red lines indicate that the training set is sent to each algorithm for 5-fold cross-validation before prediction processing. The blue line indicates that each algorithm was integrated for the test set after taking a weighted average based on the reciprocal of the RMSE.

Table S2 The pseudocode of heuristic-stacking ensemble learning.

Heuristic-stacking ensemble learning algorithm

```
Inputs:
    X_train, Y_train //Training data and labels
    All_Learners //Complete set of possible base learners
    Meta_Model //The model used to combine the predictions from the base learners
    Evaluate(Model) //Function to evaluate model performance
    Sort_Learners_By_Correlation (All_Learners) //Function to sort learners by their correlation

Initialize:
    Selected_Learners = {} //an empty set
    Removed_Learners = {} //an empty set
    Sorted_Learners = Sort_Learners_By_ Correlation (All_Learners)
    Best_Performance = -Infinity
    Continue_Search = True

Bidirectional Search:
    While Continue_Search:
```

```
Forward Search (start from most important):
    Best_Learner_Forward = NULL
    For each Learner in Sorted_Learners not in Selected_Learners:
        If Learner in Removed_Learners, continue
        Temp_Selected = Selected_Learners + {Learner}
        Performance = Evaluate(Stacking_Model(Temp_Selected, Meta_Model,
                                              X_train, Y_train))
      If Performance > Best_Performance:
          Best_Performance = Performance
          Best_Learner_Forward = Learner
    If Best_Learner_Forward is not NULL:
        Add Best_Learner_Forward to Selected_Learners

Backward Search (start from least important):
    Best_Learner_Backward = NULL
    For each Learner in reversed(Sorted_Learners) in Selected_Learners:
        Temp_Selected = Selected_Learners - {Learner}
        Performance = Evaluate(Stacking_Model(Temp_Selected, Meta_Model,
                                              X_train, Y_train))
      If Performance > Best_Performance:
          Best_Performance = Performance
          Best_Learner_Backward = Learner
    If Best_Learner_Backward is not NULL:
        Remove Best_Learner_Backward from Selected_Learners
        Add Best_Learner_Backward to Removed_Learners

Update Continue_Search:
    If no changes in Selected_Learners or all Learners are in Selected_Learners or
                                                          Removed_Learners
    set Continue_Search = False
```

Final Model:

Train the final stacking model using Selected_Learners as base learners and Meta_Model as the meta learner.
